# Growth performance, meat quality, and blood characteristics of finisher crossbred pigs fed diets supplemented with different levels of green tea (*Camellia sinensis*) by-products

**DOI:** 10.14202/vetworld.2023.27-34

**Published:** 2023-01-07

**Authors:** Nguyen Cong Oanh, Cu Thi Thien Thu, Nguyen Thi Hong, Nguyen Thi Phuong Giang, Jean-Luc Hornick, Pham Kim Dang

**Affiliations:** 1 Department of Animal Physiology and Behavior, Faculty of Animal Science, Vietnam National University of Agriculture, Trau Quy, Gialam, 131000 Hanoi, Vietnam; 2 Department of Veterinary Management of Animal Resources, FARAH Center, Faculty of Veterinary Medicine, University of Liège, Quartier Vallée 2, 4000 Liège, Belgium; 3 Central Lab, Faculty of Food Science and Technology, Vietnam National University of Agriculture, Trau Quy, Gialam, 131000 Hanoi, Vietnam

**Keywords:** blood parameter, finishing pigs, green tea, growth performance, meat quality

## Abstract

**Background and Aim:**

Dietary supplementation with green tea by-product shows special effects on animal parameters. This study aimed to assess the effects of green tea by-products (GTBP) in the diet on some blood parameters, growth performance, and carcass characteristics of finishing pigs and on meat quality, and nutritional composition of pork.

**Materials and Methods:**

One hundred and sixty crossbred pigs with an initial body weight of 65.15 ± 0.38 kg, were distributed into four dietary treatments, with four replicates of 10 pigs each. The dietary treatments were a basal diet (control diet, CON), and three experimental diets (GTBP8, GTBP16, and GTBP24) based on the CON diet supplemented with GTBP at 8, 16, and 24 g/kg of feed. The studied parameters were examined during the experimental period of 10 weeks.

**Results:**

No statistical differences in average daily feed intake, average daily gain, and feed conversion ratio were observed between the diet treatments (p > 0.05). Backfat thickness decreased (linear, p < 0.05) according to the GTBP levels but no other carcass parameters. Meat quality was not influenced by the GTBP levels (p > 0.05). However, pigs fed with GTBP had a decrease in cholesterol content and an increase in crude protein and total omega-3 content of pork compared to the CON diet (p < 0.05). Moreover, dietary supplementation with GTBP significantly decreased plasma cholesterol (p < 0.05), and trends for the decrease in low-density lipoprotein cholesterol and urea nitrogen were observed (linear, p = 0.08).

**Conclusion:**

Up to 24 g/kg GTBP in the diet for finishing pigs does not impair animal performance and makes carcass leaner with softer meat as well as positive effects on cholesterol and fatty acid metabolism. Further experiments are needed to determine the optimal levels of GTBP addition in finishing pig diet to produce higher meat quality.

## Introduction

The growth performance of animals is affected by many factors such as sex, age, breed, feeding, and antibiotics. Antibiotics used in animal feed, such as higher growth rate, prevention of pathogen diseases, and enhancement of economic efficiency [1–3]. However, due to the occurrence of antibiotic-resistant bacteria and antibiotic residues in animal products, the use of alternative sources to antibiotics in animal feed is required, particularly those from medicinal plants, which are considered safe and effective agents in disease prevention, growth promotion, and quality enhancement of animal products [4–7]. Regarding human dietary habits in meat consumption, the most consumed meat in the world is pork [8]. Therefore, preserving and enhancing the growth performance and meat quality of pigs are necessary.

Green tea (*Camellia sinensis*), a variety of tea plants from the Theaceae family, contains a great number of bioactive compounds with numerous health benefits [9], so it is largely used for medicinal goals in many countries in the world [10]. Vietnam is now the 6^th^ largest tea producer in the world, with 270 thousand tons produced per year [11]. However, a considerable amount of green tea by-products (GTBP) from the simple drying methods is presumed to be produced annually by tea producers to processing companies in Vietnam [12]. These by-products are potential sources of feed additives for animals but their exploitation is not effective. According to An *et al*. [12], GTBP from tea processing companies in Vietnam contains 35% dry matter (DM), and on a DM basis, 18% crude protein, 20% crude fiber, and 28% neutral detergent fiber. Moreover, GTBP still contains more than 200 bioactive compounds and 300 various substances showing antimutagenic, anticarcinogenic, and antioxidant activities [4].

Catechins (C), the main class of polyphenols found in green tea and GTBP, possess high biological activity. Dietary supplementation with green tea powder has been reported to improve average daily gain (ADG) and feed conversion ratio (FCR) in broiler chickens [13], goats [14], cattle [15], and fishes [16]. Moreover, dietary supplementation with green tea in animal diets decreased thiobarbituric acid reactive substances values and preserved the oxidative stability of pork and broiler meat [5, 17]. In addition, dietary supplementation with GTBP reduced cholesterol content and improved fatty acid composition in animal meat [14, 17, 18]. Offering GTBP to finishing pigs also resulted in positive effects on humoral and cell-mediated immunity [19]. Nevertheless, limited studies have been carried out to evaluate the effect of GTBP from the artisanal drying method in Vietnam on production parameters of pigs, while local beverage green tea companies produce an increased quantity of GTBP.

This study aimed to assess the effects of GTBP in the diet on some blood parameters, growth performance, and carcass characteristics of finishing pigs and on meat quality, and nutritional composition of pork.

## Materials and Methods

### Ethical approval

The research protocol was approved by the Ethics Committees on Animal Experiments, Vietnam National University of Agriculture, Vietnam (Application VNUA - 2021/05).

### Study period and location

This study was conducted from June to August 2022 at the experimental farm of the Faculty of Animal Science, Vietnam National University of Agriculture, Vietnam.

### Preparation of GTBP and composition analysis

The dried residues of buds and tea leaves were obtained from tea processing companies in Thai Nguyen province, Vietnam. The by-products were then ground into powder (GTBP). Samples were prepared for analysis according to the method of Vietnamese standards [20]. The main constituents of GTBP were analyzed for DM, crude protein, ether extract, ash, crude fiber, and neutral detergent fiber according to the association of analytical chemists’ methods [21]. The content of polyphenol was determined using the Folin–Ciocalteu method as previously described [22]. The amounts of individual Cs, including C, epicatechin (EC), epigallocatechin (EGC), EC gallate (ECG), and EGC gallate (EGCG) were analyzed using a Shimadzu LC-20A high-performance liquid chromatography system (Shimadzu, Kyoto, Japan), according to Balci and Özdemir [23].

### Experimental design, animals, and diets

A total of 160 crossbred growing pigs (80 males and 80 females, breeding: Duroc × (Landrace × Yorkshire), initial body weight: 65.15 ± 0.38 kg) were divided into four dietary treatments, balanced IBW, and gender, for 10 weeks feeding trial. Four replicate pens were assigned to each of the four treatments, with 10 pigs (five males and five females) per replicate pen. The effect of sex was not investigated in this study. Each pen (3.5 m × 4.3 m) was equipped with one automatic feeder and two automatic nipple drinkers. The temperature of pig house was around 26–28°C, while relative humidity was around 65%–85% over the entire experimental period. Each treatment was randomly allocated to one of four diets, including a basal diet (control diet, CON) and three other diets – GTBP8, GTBP16, and GTBP24 – based on the CON diet supplemented with GTBP at 8, 16, and 24 g/kg, respectively. Water and feeding were provided for *ad libitum* consumption over the entire experimental period.

Raw feed materials, including yellow maize, soybean meal, fish meal, rice bran, wheat bran, and others, were supplied and formulated by a local feed processing company. Nutrient levels of the basal diet met the recommended requirements for finishing pigs (Table-1) [24, 25].

**Table-1:** Ingredients of the basal diet [24].

Item	Basal diet
Ingredients (as-fed basis)	
Yellow maize	386
Soybean meal	102
Fish meal	20.0
Rice bran	250
Wheat bran	200
Limestone	15.0
Vitamin-mineral premi×^1^	5.00
NaCl	10.0
Farm enzyme^2^	5.00
L-lysine HCl, 98.5%	5.00
DL-methionine, 98%	2.00
Analyzed composition (% DM)	
DM	88.8
Crude protein	16.5
Ether extract	7.78
Crude ash	7.56
Crude fiber	5.15
Neutral detergent fiber	18.0
Calcium	1.26
Total phosphorus	0.90
Gross energy (MJ/kg DM)	18.6
Metabolizable energy^3^ (MJ/kg DM)	14.6
Lysine^4^	1.16
Methionine^4^	0.50

1 Premix in 1 kg: Activity enzyme, 100 g; biotin, 8 mg; coarse sand, 2%; CuSO_4_, 250–300 mg; FeSO_4_, 150–200 mg; ZnSO_4_, 250–300 mg; MnSO4, 150–200 mg; sufficient carrier for 1000 g; moisture, 10%.

2 Farm enzyme in 1 kg: *Candida tropicalis*, 10^5^–10^8^ CFU/g; *Lactobacillus acidophilus*, 10^9^–3.10^9^ CFU/g; *Saccharomyces boulardii*, 10^9^–2.10^10^ CFU/g; *Saccharomyces fibuligera*, 10^6^–10^10^ CFU/g; moisture (max), 10%.

3 Calculated ME value according to Noblet and Perez [25].

4 Nutrient data are estimated data, DM=Dry matter

### Sampling and measurements

#### Pig performance

The animals were individually weighed using an electronic scale of 300 kg (accuracy 0.01 kg) at the beginning and finishing dates of the trial. The average daily feed intake (ADFI, kg/pig/day), ADG (g/pig/day), and FCR (kg feed/kg pig weight gain) were recorded and calculated for each pig, replicate pen, and treatment over the entire period of the experiment [26].

#### Carcass traits

At the end of the trial, 48 pigs (12 pigs per treatment, two barrows, and two females per pen) were slaughtered by electrical head-only stunning followed by exsanguination. Hot carcass (kg), carcass weight (kg), killing-out percentage (%), dressing percentage (%), and backfat thickness (mm) were measured as previously described [27].

*Longissimus thoracis* muscle (LTM) samples at the middle of the 13^th^ and 14^th^ ribs were collected immediately after slaughter. Two subsamples of LTM (around 250 g) were taken from the left side of each carcass. The subsamples were distinctly weighed and placed in tight plastic bags. Then, one subsample was stored at 4°C for technological quality assessment at 24 h after slaughter. The other one was then kept at −20°C to analyze the chemical composition, cholesterol content, and total omega-3 fatty acids [28].

#### Technological quality of LTM muscle

pH values were determined at two points time (45 min and 24 h), and other parameters of LTM were determined at 24 h *postmortem*. pH values were measured using a portable pH (pH-STAR, Germany). Meat color CIE Lab values (L* a* b*) were determined using the model CR-410 Chroma Meter (Minolta, Japan) as previously described by Choi *et al*. [29]. Drip loss and drip cooking percentages were determined as previously described by Oanh *et al*. [27]. Shear forces were recorded using a Warner-Bratzler shear machine (USA) [29].

#### Chemical composition of pork

The chemical composition of LTM samples was analyzed through measures composed of DM content, crude protein content, lipid content, and total ash content, according to the AOAC method [21].

The content of cholesterol was analyzed using a Shimadzu GCMS-QP2010 gas chromatography (GC)–mass spectrometer (Shimadzu) as previously described by Derewiaka and Obiedziński [30]. The content of omega-3 fatty acids (C18:3n3, C20:3n3, C20:5n3, and C22:6n3) was measured using an Agilent 6890 plus GC equipped with a flame ionization detector (Agilent Technologies, USA), SP-2560 capillary GC column, according to the steps as previously described by Ding *et al*. [31].

#### Blood and serum analyses

One day before the end of the experiment, 24 animals (six pigs per treatment, one barrow, and one female per replicate pen) were randomly chosen for blood sampling through the jugular vein using an 18-G needle, as previously described by Oanh *et al*. [6]. Briefly, aliquots of blood samples were separately placed in both Vacutainer tubes containing K2EDTA and serum tubes. Hematology parameters, including red blood cell count (RBC), white blood cell count (WBC), hemoglobin (Hb) content, and lymphocyte percentage, were determined using the hematology analyzer ABX Pentra DX 120c (Horiba Medical, Montpellier, France). The serum tubes were centrifuged at 3000× *g* at 4°C for 15 min, and the plasma samples were transferred to plastic vials. The concentrations of aspartate aminotransferase (AST), alanine aminotransferase (ALT), bilirubin, total cholesterol, creatinine, high-density lipoprotein, low-density lipoprotein (LDL), protein, and urea nitrogen were determined using Cobas 8000 modular analyzer series (Roche, Germany).

### Statistical analysis

Experimental data were measured using the PROC MIXED procedure (version 9.4; SAS Inst. Inc., Cary, NC, USA), and diet was the fixed effect. Pens were as the experimental unit for growth performance, whereas individual pigs served as the experimental units for carcass characteristics, meat quality, chemical composition, and sensory data. Orthogonal polynomials were used to determine the linear and quadratic effects of increasing the inclusion of GTBP in diets on studied parameters. The results are shown as the least square means with a standard error of the mean. Multiple comparisons were determined using Tukey adjustment. The significance level was tested as p ≤ 0.05, while 0.05 < p < 0.10 was a trend.

## Results

### Constituents of GTBP

The GTBP product had crude protein and crude fiber close to 20%. The contents in total polyphenols were close to 20%. The EGCG proportion among total polyphenols was significantly higher than that of ECG, EGC, EC, and C (Table-2).

**Table-2:** Chemical composition and bioactive compounds of green tea by-products used in the experiment.

Item	Value
Chemical composition (% DM)	
Moisture, %	9.50
Crude protein, %	18.6
Ether extract, %	3.33
Crude fiber, %	19.8
Crude ash, %	4.68
Bioactive compound (% DM)	
Total polyphenol (%)	19.5
Total catechins (%)	14.9
Catechin	0.33
Epicatechin	0.86
Epigallocatechin	2.23
Epicatechin gallate	2.19
Epigallocatechin gallate	9.15

DM=Dry matter

### Growth performance and carcass parameters

The different inclusion levels of GTBP in the experimental diets did not influence final live body weight (FBW), ADFI, ADG, and FCR (p > 0.05). However, pigs that received the diet supplemented with 2.4% GTBP had a slightly decreasing trend in ADG in comparison with other diets (Table-3).

**Table-3:** Growth performance (LSM) of finishing pigs fed diets with different levels of green tea by-products.

Item	Dietary treatment^1^				SEM	p-value	
	
	CON	GTBP8	GTBP16	GTBP24		Linear	Quadratic
Number of pigs	40	40	40	40			
IBW (kg)	65.11	65.22	65.11	65.17	0.75	0.98	0.97
FBW (kg)	113.8	114.6	114.0	111.3	1.38	0.20	0.21
ADFI (kg/d)	2.15	2.19	2.19	2.10	0.05	0.47	0.27
ADG (g/d)	695	705	698	658	16.7	0.13	0.15
FCR (kg/kg)	3.11	3.10	3.14	3.20	0.13	0.62	0.81

1 CON: Control diet; GTBP8, GTBP16, and GTBP24 based on CON supplemented with green tea by-products at 0.8, 1.6, and 2.4%, d=day, g=gram, IBW=Initial live body weight, FBW=Final live body weight, ADG=Average daily weight gain, ADFI=Average daily dry matter feed intake, FCR=Feed conversion ratio, LSM=Least square means

Dietary supplementation of GTBP in the diets did not influence killing-out percentage and dressing percentage (p > 0.05). However, a significant decrease (linear, p = 0.04) in back fat thickness (BFT) was found for pigs fed diets with increasing GTBP levels, with the lowest value of BFT when the diet was supplemented with 2.4% GTBP (Table-4).

**Table-4:** Carcass traits (LSM) of finishing pigs fed diets with different levels of green tea by-products.

Item	Dietary treatment^1^				SEM	p-value	
	
	CON	GTBP8	GTBP16	GTBP24		Linear	Quadratic
Number of pig	8	8	8	8			
Final body weight, kg	113.0	114.8	114.2	111.8	1.75	0.60	0.25
Killing-out percentage, %	80.2	80.9	80.6	80.2	0.45	0.80	0.23
Dressing percentage, %	70.3	71.4	70.6	70.4	0.49	0.75	0.18
Backfat thickness, mm	18.9	16.6	16.4	16.1	0.91	0.04	0.28

1 CON: Control diet; GTBP8, GTBP16, and GTBP24 based on CON supplemented with green tea by-products at 0.8, 1.6, and 2.4%; six slaughtered pigs per each experimental group, LSM=Least square means, SEM=Standard error of the mean

### Technological quality of pig meat

The pH values (pH45 and pH24), drip losses (DL24), and cooking loss (CL24) of LTM were not significantly different (linear, p ≥ 0.39) between pigs fed the diets with the inclusion of GTBP and the CON (Table-5). The shear forces (SF24) of LTM tended to decrease (linear, p = 0.05) in response to the addition of GTBP when compared to the control group. Meat lightness (L*24) did not differ between the groups, but redness (a*) and yellowness (b*) tended to increase trend in pigs offered GTBP (linear, p ≤ 0.08).

**Table-5:** Technological parameters of pork (LSM) in finishing pigs fed diets with different levels of green tea by-products.

Items	Dietary treatment^1^				SEM	p-value	
	
	CON	GTBP8	GTBP16	GTBP24		Linear	Quadratic
Number of pig	8	8	8	8			
pH at 45 min	6.31	6.36	6.34	6.34	0.04	0.83	0.54
pH at 24 h	5.52	5.51	5.52	5.49	0.03	0.52	0.86
Drip loss at 24 h (%)	1.30	1.10	1.22	1.25	0.25	0.98	0.63
Shear force at 24 h (N)	39.2	36.3	35.4	33.5	1.80	0.05	0.78
Lightness at 24 h (L*)	54.4	55.0	55.5	56.1	1.45	0.39	0.99
Redness at 24 h (a*)	11.9	12.7	13.4	13.4	0.45	0.06	0.21
Yellowness at 24 h (b*)	5.16	6.10	6.16	6.13	0.34	0.08	0.20

1 CON: Control diet; GTBP8, GTBP16, and GTBP24 based on CON supplemented with green tea by-products at 0.8, 1.6, and 2.4%, respectively. LSM=Least square means, SEM=Standard error of the mean; CIE L*, a*, b* (L=black (0) to white (100), a=green (-) to red (+) color scale, b=blue (-) to yellow (+) color scale) at 24 h

### Chemical composition of pig meat

Pigs fed diets supplemented with GTBP tended to have increased crude protein content (linear, p = 0.04; quadratic, p = 0.02) and decreased lipid content in LTM meat (quadratic, p = 0.01), but DM and ash content were not changed. A decreased trend in cholesterol content (linear, p = 0.08; quadratic, p = 0.01) and an increase in total omega-3 fatty acid content (C18:3n3, C20:3n3, C20:5n3, and C22:6n3) (both linear and quadratic p = 0.01) in LTM meat were observed in pigs received GTBP in comparison to CON (Table-6).

**Table-6:** Nutritional composition of pork (LSM) of finishing pigs fed diets with different levels of green tea by-products.

Items	Dietary treatment^1^				SEM	p-value	
	
	CON	GTBP8	GTBP16	GTBP24		Linear	Quadratic
Number of pig	8	8	8	8			
Dry matter, %	26.9	27.3	27.7	27.4	0.31	0.28	0.14
Crude protein, %	22.2^b^	23.2^a^	23.0^ab^	23.7^a^	0.26	0.04	0.02
Lipids, %	2.57^a^	2.42^a^	2.30^ab^	1.98^b^	0.09	0.75	0.01
Ash, %	1.40	1.43	1.35	1.43	0.03	0.92	0.39
Cholesterol, mg/100 g	59.1^a^	54.5^a^	42.8^b^	39.2^b^	1.24	0.08	0.01
Total omega-3, mg/100 g	17.0^d^	32.5^c^	40.0^b^	54.4^a^	1.55	0.01	0.01

1 CON: Control diet; GTBP8, GTBP16, and GTBP24 based on CON supplemented with green tea by-products at 0.8, 1.6, and 2.4%, respectively. Mean values in the same row with different letters^(a,b,c)^ are significantly different (p < 0.05), SEM=Standard error of the mean

### Blood parameters

The inclusion of GTBP did not induce a significant effect on blood parameters, including WBC, RBC, Hb, AST, ALT, bilirubin, protein, and creatinine (Table-7). However, plasma cholesterol content decreased with increasing GTBP levels in the diets (linear, p = 0.02). Moreover, a trend for a decrease in blood LDL-cholesterol and urea nitrogen was observed in pigs fed diets with increasing GTBP (linear, p = 0.08).

**Table-7:** Blood parameters (LSM) of finishing pigs fed diets with different levels of green tea by-products.

Items	Dietary treatment^1^				SEM	p-value	
	
	CON	GTBP8	GTBP16	GTBP24		Linear	Quadratic
Number of test pigs	8	8	8	8			
White blood cell (G/L)	14.5	17.2	15.5	17.7	1.15	0.15	0.83
Red blood cell (T/L)	6.57	7.06	6.65	6.48	0.36	0.68	0.36
Hemoglobin (g/dL)	10.3	11.3	10.9	11.8	0.77	0.27	0.92
AST, U/L	56.2	69.5	63.4	59.2	9.88	0.95	0.39
ALT, U/L	54.3	65.8	58.2	55.2	5.65	0.85	0.22
Bilirubin (mmol/L)	0.73	0.78	0.74	0.84	0.13	0.65	0.85
Protein (g/L)	70.6	70.7	72.6	71.2	1.52	0.60	0.60
Creatinine (mmol/L)	108	115	120	133	7.65	0.65	0.85
Total cholesterol (mmol/L)	2.62	2.52	2.38	2.09	0.14	0.02	0.53
High-density lipoprotein (mmol/L)	1.08	1.13	1.04	1.05	0.05	0.34	0.66
Low-density lipoprotein (mmol/L)	1.21	1.07	1.06	0.88	0.12	0.08	0.88
Urea nitrogen (mmol/L)	5.45	4.32	5.08	4.17	0.36	0.08	0.77

LSM=Least squares means, SEM=Standard error of the mean, AST=Aspartate aminotransferase, ALT=Alanine aminotransferase

## Discussion

This study aimed to assess the effects of GTBP in the diet on some blood parameters, growth performance, and carcass characteristics of finishing pigs and on meat quality, and nutritional composition of pork. The chemical composition of GTBP was in the same range as values reported for similar by-products [12, 27]. Total phenolic content in the experimental GTBP was in the range of 13.2%–21.7% DM, as reported by Khoa *et al*. [32]. Balci and Özdemir [23] reported a total polyphenol concentration between 6.8% and 13.1% DM, which is lower as compared to our results. This may be related to the tea varieties, geographical areas, time of harvesting and leaf age, leaf buds, processing methods, and extraction conditions [32, 33]. Moreover, EGCG content was the major compound representing 61.4% of the total Cs in GTBP. According to Zaveri [34], EGCG was the most abundant C in green tea, accounting for 65% of the total Cs constituent. Khoa *et al*. [32] reported that the content of EGCG in green tea was found in the range of 52%–63%. Many of the health benefits of green tea for humans and animals relate to Cs, particularly EGCG content [23, 35].

In this study, supplementation of finishing pigs with diet inclusions of GTBP was evaluated through production parameters. Growth performance, including FBW, ADG, and FCR was not affected by the dietary supplementation with GTBP at 0.8, 1.6, and 2.4% when compared to the control group. These results are in line with the previous studies [4, 19], which obtained similar data of FBW and ADG by adding up to 2% GTBP in finisher pig diets. However, Hossain *et al*. [4] reported that significantly higher values of ADFI and FCR were found in a finisher pig diet supplemented with GTBP at 2% compared to a CON. In addition, dietary supplementation at higher GTBP levels was negatively related to body weight gain in pigs and broilers [18, 36], probably due to great tannin concentration in GTBP leading to the inhibition of protein digestion and also to the high-fiber content in GTBP. The contradictory results regarding ADFI and FCR responses to GTBP could be differences in pig breeds, animals’ age, or experiment lasting.

In this study, different diets did not influence the killing-out and dressing percentage. However, BFT decreased with increased supplementation of GBTP, which is consistent with the previous studies on mice [37, 38]. This is probably associated with Cs contents of GTBP by interference in digestive lipase activity, inhibition of synthesis, and upregulation of β-oxidation of fatty acids in animal liver, thereby reducing BFT accumulation in animals [4, 36].

This study found that dietary supplementation of GTBP did not change pH values at 45 min and 24 h. The technological parameters of LTM meat in our work were classified as normal meat as described by Lengerken and Pfeiffer [39] and Monin [40]. Moreover, the shear force of pig meat decreased with increasing supplementation levels of GTBP, which is consistent with a similar study confirmed by Hossain *et al*. [4]. Smaller shear force results in softer meat and better taste. Therefore, dietary supplementation of GBTP improved meat quality which meets the demands of today’s market. Dietary GTBP supplementation did not significantly influence the surface color of LTM meat. However, meat color in redness (a*) and yellowness (b*) of LTM meat tended to increase with increasing inclusion of GTBP, which is consistent with the previous results found by Uuganbayar *et al*. [41], who stated that dietary supplementation with GTBP at 2% increased redness and yellowness values of animal meat. This phenomenon could be due to the pigments of GTBP that percolate directly in meat [4].

The pork from pigs fed diets supplemented with GTBP had a higher protein content relative to the CON, which is consistent with a previous study by Flores-Mancheno *et al*. [42]. Dietary supplementation with GTBP in the finishing diets significantly decreased the cholesterol content of LM meat, which may have been due to the high polyphenol, particularly EGCG, that could form insoluble complexes with cholesterol in the gastrointestinal tract and inhibit the absorption of endogenous and exogenous cholesterol in the intestine of animals [43, 44].

In this study, a significant reduction of blood cholesterol content in finishing pigs when their diets were supplemented with GTBP is similar to the results obtained from the previous studies [13, 45] that found that the blood cholesterol content in broilers was reduced when their diets were supplemented with green tea powder up to 1.5%. This could be due to the GTBP inhibiting the activity of β-hydroxy β-methylglutaryl-CoA reductase as a rate-limiting enzyme in the cholesterol biosynthesis pathway [46].

## Conclusion

In Vietnamese finishing pigs, the supplementation with GTBP up to 2.4% into the diets appears to have strong effects on lipid metabolism by reducing the total amount of body lipids without affecting animal performance. Its effects on meat technological parameters are unclear. Further experiments need to be carried out to determine the optimal levels of GTBP addition in the finishing pig diet to produce higher meat quality.

## Authors’ Contributions

PKD, NCO, and JH: Conceived and designed the study. NCO, NTPG, and CTTT: Conducted the trial and collected the samples. NCO and NTH: Analyzed the pork and blood samples. NCO and JH: Analyzed the data. PKD, NCO, and CTTT: Revised the manuscript. All authors have read and approved the final manuscript.

## References

[1] Barton M.D (2000). Antibiotic use in animal feed and its impact on human health. Nutr. Res. Rev.

[2] Çilek S, Gotoh T (2014). Effects of dam age, lamb gender, and singleton or twin status on body size of Malya lambs in middle Anatolia, Turkey. J. Fac. Agric. Kyushu Univ.

[3] Li J (2017). Current status and prospects for in-feed antibiotics in the different stages of pork production-a review. Asian Australas. J. Anim. Sci.

[4] Hossain M.E, Ko S.Y, Yang C.J (2012). Dietary supplementation of green tea by-products on growth performance, meat quality, blood parameters and immunity in finishing pigs. J. Med. Plants Res.

[5] Ko S.Y, Bae I.H, Yee S.T, Lee S.S, Uuganbayar D, Oh J.I, Yang C.J (2008). Comparison of the effect of green tea by-product and green tea probiotics on the growth performance, meat quality, and immune response of finishing pigs. Asian Australas. J. Anim. Sci.

[6] Oanh N.C, Lam T.Q, Tien N.D, Hornick J.L, Ton V.D (2021). Effects of medicinal plants mixture on growth performance, nutrient digestibility, blood profiles, and fecal microbiota in growing pigs. Vet. World.

[7] Rao R.R, Platel K, Srinivasan K (2003). *In vitro* influence of spices and spice-active principles on digestive enzymes of rat pancreas and small intestine. Nahrung.

[8] Qi J, Li Y, Zhang C, Wang C, Wang J, Guo W, Wang S (2021). Geographic origin discrimination of pork from different Chinese regions using mineral elements analysis assisted by machine learning techniques. Food Chem.

[9] Perumalla A.V, Hettiarachchy N.S (2011). Green tea and grape seed extracts-potential applications in food safety and quality. Food Res. Int.

[10] Graham H.N (1992). Green tea composition, consumption, and polyphenol chemistry. Prev. Med.

[11] Phuoc H.P, Huynh T.T, Le T.T (2020). Research on microwave treatment for fixation of polyphenol oxidase in processing Oolong tea in Vietnam. Southeast Asian J. Sci.

[12] An L.T, Thang C.M, Cuong P.K, Hiep T (2020). Assessing the source of tea by-products as supplementary feed in cattle production. J. Anim. Sci. Technol.

[13] Biswas A.H, Wakita M (2001). Effect of dietary Japanese green tea powder supplementation on feed utilization and carcass profiles in broilers. J. Poult. Sci.

[14] Ahmed S.T, Lee J.W, Mun H.S, Yang C.J (2015). Effects of supplementation with green tea by-products on growth performance, meat quality, blood metabolites and immune cell proliferation in goats. J. Anim. Physiol. Anim. Nutr (Berl).

[15] Sarker M.S.K, Ko S.Y, Lee S.M, Kim G.M, Choi J.K, Yang C.J (2010). Effect of different feed additives on growth performance and blood profiles of Korean Hanwoo calves. Asian Australas. J. Anim. Sci.

[16] Kono M, Furukawa K, Sagesaka Y.M, Nakagawa K, Fujimoto K (2000). Effect of green tea grounds as dietary supplements on cultured yellowtail and ayu. J. Jpn. Soc. Food Sci. Technol.

[17] Yang C.J, Yang I.Y, Oh D.H, Bae I.H, Cho S.G, Kong I.G, Uuganbayar D, Nou I.S, Choi K.S (2003). Effect of green tea by-product on performance and body composition in broiler chicks. Asian Australas. J. Anim. Sci.

[18] Suzuki K, Kadowaki H, Hino M, Tamura K (2002). The influence of green tea in pig feed on meat production and quality. Nihon Yoton Gakkaishi.

[19] Ko S.Y, Yang C.J (2008). Effect of green tea probiotics on the growth performance, meat quality and immune response in finishing pigs. Asian Australas. J. Anim. Sci.

[20] TCVN (2007). Vietnamese Standard (TCVN 5609:2007):Tea-Sampling.

[21] Association of Official Analytical Chemists (1990). Official Methods of Analysis.

[22] Josipović A, Sudar R, Sudarić A, Jurković V, Kocar M.M, Kulundžić A.M (2016). Total phenolic and total flavonoid content variability of soybean genotypes in Eastern Croatia. Croat. J. Food Sci. Technol.

[23] Balci F, Özdemir F (2016). Influence of shooting period and extraction conditions on bioactive compounds in Turkish green tea. Food Sci. Technol.

[24] National Research Council (2012). Nutrient Requirements of Swine.

[25] Noblet J, Perez J.M (1993). Prediction of digestibility of nutrients and energy values of pig diets from chemical analysis. J. Anim. Sci.

[26] TCVN (1984). Method for Estimating a Pig-carcass Slaughter.

[27] Oanh N.C, Bernard T, Kim D.P, Duc L.D, Nassim M, Thi H.N, Hoang T.N, Georges D, Jerome B, Dinh T.V, Hornick J.L (2019). Growth performance, carcass quality characteristics and colonic microbiota profiles in finishing pigs fed diets with different inclusion levels of rice distillers'by-product. Anim. Sci. J.

[28] Cong O.N, Huyen N, Kim D.P, Dinh T.V, Hornick J.L (2021). Growth performance, carcass traits, meat quality and composition in pigs fed diets supplemented with medicinal plants (*Bindens pilosa* L., *Urena lobata* L. and *Ramulus cinnamomi*) powder. J. Anim. Feed Sci.

[29] Choi J.S, Lee H.J, Jin S.K, Choi Y.I, Lee J.J (2014). Comparison of Carcass characteristics and meat quality between Duroc and crossbred Pigs. Korean J. Food Sci. Anim. Resour.

[30] Derewiaka D, Obiedziński M (2010). Cholesterol oxides content in selected animal products determined by GC-MS. Eur. J. Lipid Sci. Technol.

[31] Ding X, Yang C.W, Yang Z.B (2017). Effects of Star anise (*Illicium verum* hook.f.), essential oil, and leavings on growth performance, serum, and liver antioxidant status of broiler chickens. J. Appl. Poult. Res.

[32] Khoa G.T, Hai N.T, Manh N.X, Thuy N.T.B, Nghia P.D, Huong P.T, Duez P (2013). Effects of raw material types on the chemical composition of Trung Du tea variety (*Camellia sinensis* var. sinensis). J. Sci. Dev.

[33] Prasanth M.I, Sivamaruthi B.S, Chaiyasut C, Tencomnao T (2019). A review of the role of green tea (*Camellia sinensis*) in antiphotoaging, stress resistance, neuroprotection, and autophagy. Nutrients.

[34] Zaveri N.T (2006). Green tea and its polyphenolic catechins:Medicinal uses in cancer and noncancer applications. Life Sci.

[35] Chacko S.M, Thambi P.T, Kuttan R, Nishigaki I (2010). Beneficial effects of green tea:A literature review. Chin. Med.

[36] Sayama K, Lin S, Zheng G, Oguni I (2000). Effects of green tea on growth, food utilization and lipid metabolism in mice. In Vivo.

[37] Sugiura C, Nishimatsu S, Moriyama T, Ozasa S, Kawada T, Sayama K (2012). Catechins and caffeine inhibit fat accumulation in mice through the improvement of hepatic lipid metabolism. J. Obes.

[38] Zheng G, Sayama K, Okubo T, Juneja L.R, Oguni I (2004). Anti-obesity effects of three major components of green tea, catechins, caffeine and theanine, in mice. In Vivo.

[39] Lengerken G.V, Pfeiffer H (1987). Status and development tendencies of the application of methods for the detection of stress sensitivity and meat quality in pigs (in German:Stand und Entwicklungstendezen der Anwendung von Methoden zur Erkennung der Stressempfindlichkeit und Fleischqualitaet beim Schwein). Internatonal Symposium about pig breeding (Zur Schweinezucht). Leipzig (Germany).

[40] Monin G (2003). Pig slaughtering and carcass and meat quality (In French:Abattage des porcs et qualités des carcasses et des viandes). Prod. Anim.

[41] Uuganbayar D, Bae I.H, Choi K.S, Shin I.S, Firman J.D, Yang C.J (2005). Effects of green tea powder on laying performance and egg quality in laying hens. Asian Australas. J. Anim. Sci.

[42] Flores-Mancheno C.I, Duarte C, Salgado-Tello I.P (2017). Characterization of the Guinea pig (*Cavia porcellus*) meat for fermented sausage preparation. Rev. Cienc. Agric.

[43] Ahmed S.T, Mun H.S, Islam M.M, Ko S.Y, Yang C.J (2016). Effects of dietary natural and fermented herb combination on growth performance, carcass traits and meat quality in grower-finisher pigs. Meat Sci.

[44] Rao A.V, Gurfinkel D.M (2000). The bioactivity of saponins:Triterpenoid and steroidal glycosides. Drug Metabol. Drug Interact.

[45] Liu W, Rouzmehr F, Seidavi A (2018). Effect of amount and duration of waste green tea powder on the growth performance, carcass characteristics, blood parameters, and lipid metabolites of growing broilers. Environ. Sci. Pollut. Res.

[46] Mahdavi A, Bagherniya M, Fakheran O, Reiner Ž, Xu S, Sahebkar A (2020). Medicinal plants and bioactive natural compounds as inhibitors of HMG-CoA reductase:A literature review. Biofactors.

